# Chronic kidney disease prevalence and associated risk factors in hypertensive adults at Burao general hospital, Burao City, Somaliland: a cross-sectional study

**DOI:** 10.3389/fcvm.2025.1503233

**Published:** 2025-08-07

**Authors:** Dek Kahin Yosef, Yusuf Ahmed Ali

**Affiliations:** ^1^ School of Postgraduate Studies and Research, Faculty of Medicine and Health Science, University of Burao, Burao, Somaliland; ^2^ School of Medicine, Faculty of Medicine and Health Science, University of Burao, Burao, Somaliland

**Keywords:** chronic kidney disease, hypertension, Burao, Somaliland, proteinuria

## Abstract

**Background:**

Chronic kidney disease (CKD) is a significant global health challenge, particularly in low- and middle-income countries where hypertension is a prominent risk factor. This study aimed to assess the prevalence of CKD and its associated risk factors among hypertensive adults at the Burao General Hospital in Somaliland, Somalia.

**Methods:**

A cross-sectional study was conducted from January to July 2024, enrolling 262 hypertensive adults using consecutive sampling. Data were collected using structured questionnaires addressing sociodemographic factors, clinical characteristics, and lifestyle choices. Proteinuria levels and estimated glomerular filtration rates were assessed to confirm CKD diagnosis.

**Results:**

The prevalence of CKD among hypertensive patients was 52.67% (95% CI: 46.6%–58.7%). Significant associations were observed between CKD and factors such as age, proteinuria, diabetes, blood pressure control, and body mass index (BMI). Specifically, proteinuria was strongly linked to CKD (AOR: 10.72, 95% CI: 5.74–20.04). Individuals aged 34–41 years (AOR: 3.39, 95% CI: 0.99–11.54) and those classified as overweight (AOR: 3.37, 95% CI: 1.65–6.88) were at greater risk for CKD.

**Conclusion:**

The findings highlight a critical association between hypertension and CKD, emphasizing the need for targeted interventions to effectively manage hypertension and address modifiable risk factors. Understanding these relationships is vital for developing healthcare policies aimed at reducing CKD prevalence among adults in Burao City, Somaliland.

## 1 Background

Chronic kidney disease (CKD) is a significant global health issue, particularly in low- and middle-income countries, where access to healthcare can be limited (1, 2). One of the leading risk factors for CKD is hypertension, which greatly increases the risk of both kidney and cardiovascular diseases (3, 4).

The correlation between hypertension and CKD has been well established in the literature (5–7). Epidemiological studies have consistently shown that hypertension is both a cause and consequence of CKD, creating a vicious cycle of kidney damage and worsening of blood pressure control (3, 8). A large meta-analysis of cohort studies found that individuals with hypertension had a 1.81 times higher risk of developing CKD than those without hypertension (95% CI: 1.47–2.23) (9–11). Additionally, clinical trials have demonstrated that intensive blood pressure control can slow CKD progression (12). The SPRINT trial showed that targeting a systolic blood pressure of <120 mmHg, compared to <140 mmHg, reduced the risk of CKD progression by 32% in non-diabetic adults (13, 14).

The prevalence of hypertension is notably high in Burao and Hargeisa City, Somaliland; however, its impact on kidney health has not been thoroughly examined (15). While there is evidence of a clear link between hypertension and CKD globally, few studies have focused specifically on adults in this region, highlighting a critical gap in our understanding (16, 17).

This study investigated the effect of hypertension on CKD among adults visiting Burao General Hospital, specifically examining associated factors such as age, diabetes, protein levels in urine, and body mass index. We hypothesized that uncontrolled hypertension is linked to a higher risk of CKD, particularly in patients with additional health. Understanding these relationships is essential for developing effective strategies to reduce the impact of CKD on individuals with high blood pressure in Burao.

The findings of this study are expected to provide valuable insights and inform healthcare policies aimed at improving kidney health in Somaliland. Therefore, this study aimed to explore the prevalence of hypertension among adults with CKD and to assess the impact of various associated factors in Burao General Hospital, Burao City, Somaliland, and Somalia.

## 2 Materials and Methods

### 2.1 Study area

This cross-sectional study was conducted at the Burao General Hospital, located in Burao City, Somaliland, Somalia. The hospital is the primary healthcare provider in the Togdheer region, serving a population of over 400,000 (18) Burao General Hospital offers comprehensive medical services, including diagnostic and treatment facilities for hypertension and chronic kidney disease (CKD), making it an ideal setting for this study (19, 20). Given the hospital's strategic location and role as a referral center, it manages a significant number of patients with hypertensive and kidney-related conditions (20). This study focused on adults visiting the hospital, representing a mix of urban and rural populations, and aimed to explore the prevalence and impact of hypertension on CKD.

### 2.2 Study design and period

The study was conducted from January to July 2024 using a hospital-based cross-sectional study design.

### 2.3 Study population and sample size determination

The source population for this study consisted of adult patients with chronic kidney disease who had hypertension in Burao City, Somaliland. The study population included all individuals diagnosed with chronic kidney disease presenting in Burao City. The overall sample size determined for this study was 262 participants based on the prevalence of chronic kidney disease among hypertensive patients in Ethiopia.

### 2.4 Eligibility criteria

#### 2.4.1 Inclusion criteria

Adult hypertensive patients aged 18 years or older attending Burao General Hospital between January and July 2024, irrespective of eGFR values were includes.

#### 2.4.2 Exclusion criteria

In contrast, pregnant patients with hypertension during the study period and individuals who developed the outcome of interest prior to or at the start of the follow-up period were excluded. Additionally, patients with an eGFR < 60 ml/min/1.73 m^2^ were also not considered for inclusion in the study.

### 2.5 Sampling techniques

A consecutive sampling technique was implemented, systematically incorporating each patient into the study to ensure comprehensive representation.

### 2.6 Data collection instrument and techniques

This study used a structured questionnaire for face-to-face interviews and patient chart review. The questionnaire was initially developed in English, translated into Somali, and back-translated into English to ensure accuracy. It covered sociodemographic information, clinical characteristics, and lifestyle-related factors associated with the prevalence of chronic kidney disease among patients at Burao General Hospital, Somaliland. Data collection was carried out by four students under the supervision of one supervisor, and interviews were conducted directly with the respondents. Data quality control was ensured through a 5% pre-test, leading to minor modifications based on expert suggestions. The supervisor checked the data daily for completeness and the principal investigator reviewed the data before and after administration. Backup data were stored on an external hard drive and each questionnaire was coded to prevent duplication and errors.

### 2.7 Measurement tools

Data on body mass index (BMI) and blood pressure control status were collected and analyzed as categorical variables. BMI was classified as normal vs. overweight/obese, while blood pressure control was classified as controlled (<140/90 mmHg) vs. uncontrolled (≥140/90 mmHg), as per the patient records. Therefore, mean values and standard deviations were not calculated for these variables. For the urine dipstick test, the patient was asked to provide a random midstream urine sample in a 50 ml container before admission for laboratory analysis. The samples were tested for protein and creatinine levels using a Uriplus 900 urinalysis strip. The manufacturer's grades for proteinuria are as follows:

• 0: Absent
• Traces: 15–30 mg/dl
• 1+: 30–100 mg/dl
• 2+: 100–300 mg/dl
• 3+: 300–1,000 mg/dl
• 4+: Greater than 1,000 mg/dl

Estimated glomerular filtration rate (eGFR) was used to evaluate kidney function by estimating the volume of blood filtered by the kidneys over time. This can be calculated using formulae such as the Cockcroft-Gault equation (adjusted for body surface area [BSA]):

(140−Age)×Weight(kg)×(0.86forfemales)×1.73/(72×Serumcreatinine(mg/dL)×BSA)

BSA can be calculated using the Mosteller formula, as follows:

BSA=Weight(kg)×Height(cm)/60

here, weight was measured in kilograms and height in centimeters.

### 2.8 Data processing and analysis

The data were coded, entered, and cleaned using EpiData Manager version 4.2, then exported to STATA version 17 for further analysis. Chronic kidney disease was confirmed through a review of medical charts at hospitals. Baseline demographic, clinical, laboratory, and social factors were considered independent variables in the analysis. A binary logistic regression model was used to assess the relationship between the outcomes and these independent variables. Variables with a *P*-value of less than 0.25 in the bivariate analysis were included in the multivariable binary logistic regression. Odds ratios with 95% confidence intervals were calculated, and a *P*-value below 0.05 was considered indicative of a significant association. Multicollinearity was assessed using the Variance Inflation Factor (VIF). A threshold of 2.5 was used to indicate potential multicollinearity. All variables included in the multivariate logistic regression model had VIF values well below this cutoff.

### 2.9 Variables

#### 2.9.1 Dependent variable

Chronic kidney disease.

#### 2.9.2 Independent variables

Sociodemographic factors included age, sex, education level, occupation, income, and marital status. Clinical characteristics: blood pressure status, baseline diastolic and systolic blood pressure, hypertension stage, type of hypertension, number of medications, duration of medication, types of medications, presence of diabetes mellitus, dyslipidemia, and obesity. Lifestyle factors: Smoking and alcohol consumption.

### 2.10 Operational definitions

#### 2.10.1 Alcohol exposure

Participants were considered alcohol consumers if their medical records indicated any intake of alcoholic beverages.

#### 2.10.2 Chronic kidney disease (CKD)

Defined according to KDIGO guidelines as either an estimated glomerular filtration rate (eGFR) < 60 ml/min/1.73 m^2^, or evidence of kidney damage (proteinuria ≥1+ on urine dipstick) persisting for at least 3 months, documented in medical records (21, 22).

#### 2.10.3 Diabetes mellitus

Diabetes mellitus is defined as a disorder in which the body does not produce sufficient insulin or fails to respond to insulin effectively, leading to consistently elevated blood sugar (glucose) levels.

#### 2.10.4 Hypertension

Hypertension was identified in participants with a documented diagnosis of elevated blood pressure (BP ≥ 140/90 mmHg) or in those on antihypertensive medications. The target BP control was defined as BP < 140/90 mmHg for non-diabetic patients and BP < 130/80 mmHg for diabetic patients (23).

## 3 Result

### 3.1 Socio demographic characteristics

The demographic data indicated that the majority of participants were aged 34–41 years (28.24%), followed by those aged 27–33 (14.50%) and 42–48 (19.85%). The sample consisted of 47.33% males and 52.67% females. In terms of marital status, most participants were married (79.01%), with smaller percentages identifying single (8.78%), divorced (9.92%), and widowed (2.29%) (Table 1).

**Table 1:** Sociodemographic characteristics of the participants (*N* = 262).

Variables	Category	Frequency	Percentage
Age	20–26	19	7.25%
	27–33	38	14.50%
	34–41	74	28.24%
	42–48	52	19.85%
	49–55	40	15.27%
	<56	39	14.89%
Sex	Male	124	47.33%
	Female	138	52.67%
Marital statues	Single	23	8.78%
	Married	207	79.01%
	Divorced	26	9.92%
	Widow	6	2.29%

### 3.2 Clinical characteristics and lifestyle factor

The findings of the study revealed that a significant majority of the participants (72.14%) had systolic hypertension, primarily classified as primary hypertension (64.89%). Most individuals (61.45%) were prescribed one antihypertensive medication. Proteinuria was detected in 42.75% of the participants, whereas diabetes mellitus was present in 39.08%. Dyslipidemia affects 28.24% of the population. The majority of the participants (76.34%) had a normal BMI, while 23.66% were classified as overweight or obese. Regarding blood pressure control, 84.56% of the participants had controlled hypertension and 15.44% had uncontrolled hypertension. As BMI and blood pressure were recorded in categorical formats, the mean and standard deviation values were not calculated for these parameters. Additionally, 45.5% of the participants reported being smokers, indicating a significant prevalence of these clinical factors (Table 2).

**Table 2:** Clinical characteristics and lifestyle factor.

Variables	Category	Frequency	Percentage
Types of hypertensions	Systolic HTN	189	72.14%
	Mixed	73	27.86%
Classification of hypertension	Primary	170	64.89%
	Secondary	92	35.11%
Number of drugs	One	161	61.45%
	Two	72	27.48%
	Three and above	29	11.07%
Proteinuria	Yes	112	42.75%
	No	150	57.25%
Diabetic mellitus	Yes	102	39.08%
	No	159	60.92%
Dyslipidemia	Yes	74	28.24%
	No	188	71.76%
Blood pressure status	Controlled	219	84.56%
	Uncontrolled	40	15.44%
Body mass index	Normal	200	76.34%
	Overweight/obese	62	23.66%
Smoking	Yes	120	45.5%
	No	144	54.5%

### 3.3 The prevalence of chronic kidney diseases

The overall prevalence of CKD among hypertensive patients was found to be 52.67% (95% CI: 46.6%–58.7%), which included those with reduced kidney function and/or evidence of kidney damage (proteinuria).

### 3.4 Factors associated with chronic kidney diseases

This study examined the factors associated with chronic kidney disease (CKD) among adult hypertensive patients at the Burao General Hospital in 2024. A total of 124 patients diagnosed with CKD were compared with 153 hypertensive patients without CKD. Bivariate analysis revealed several notable associations; however, the focus was on the significant factors determined through multivariable logistic regression analysis (AOR). The age group of 34–41 years showed a significant association with CKD, with an adjusted odds ratio of 3.39 (95% CI: 0.99–11.54; *P* = 0.05), indicating that individuals in this age range are more likely to develop CKD compared to the reference age group (20–26 years). However, this association should be interpreted with caution given the borderline statistical significance (*P* = 0.05) and wide confidence interval (0.99–11.54).

The presence of proteinuria was strongly associated with CKD, yielding an AOR of 10.72 (95% CI: 5.74–20.04; *P* = 0.00), underscoring the critical role of proteinuria as a risk factor for CKD in hypertensive patients. Additionally, diabetic patients had a significant association with CKD, with a COR of 9.74 (95% CI: 5.49–17.29; *P* = 0.00), highlighting the exacerbating effect of diabetes on renal health in hypertensive individuals. Uncontrolled blood pressure was associated with a decreased likelihood of CKD (AOR: 0.22; 95% CI: 0.09–0.53; *P* = 0.00), suggesting that effective management of hypertension may mitigate the risk of developing CKD.

**Figure 1:**
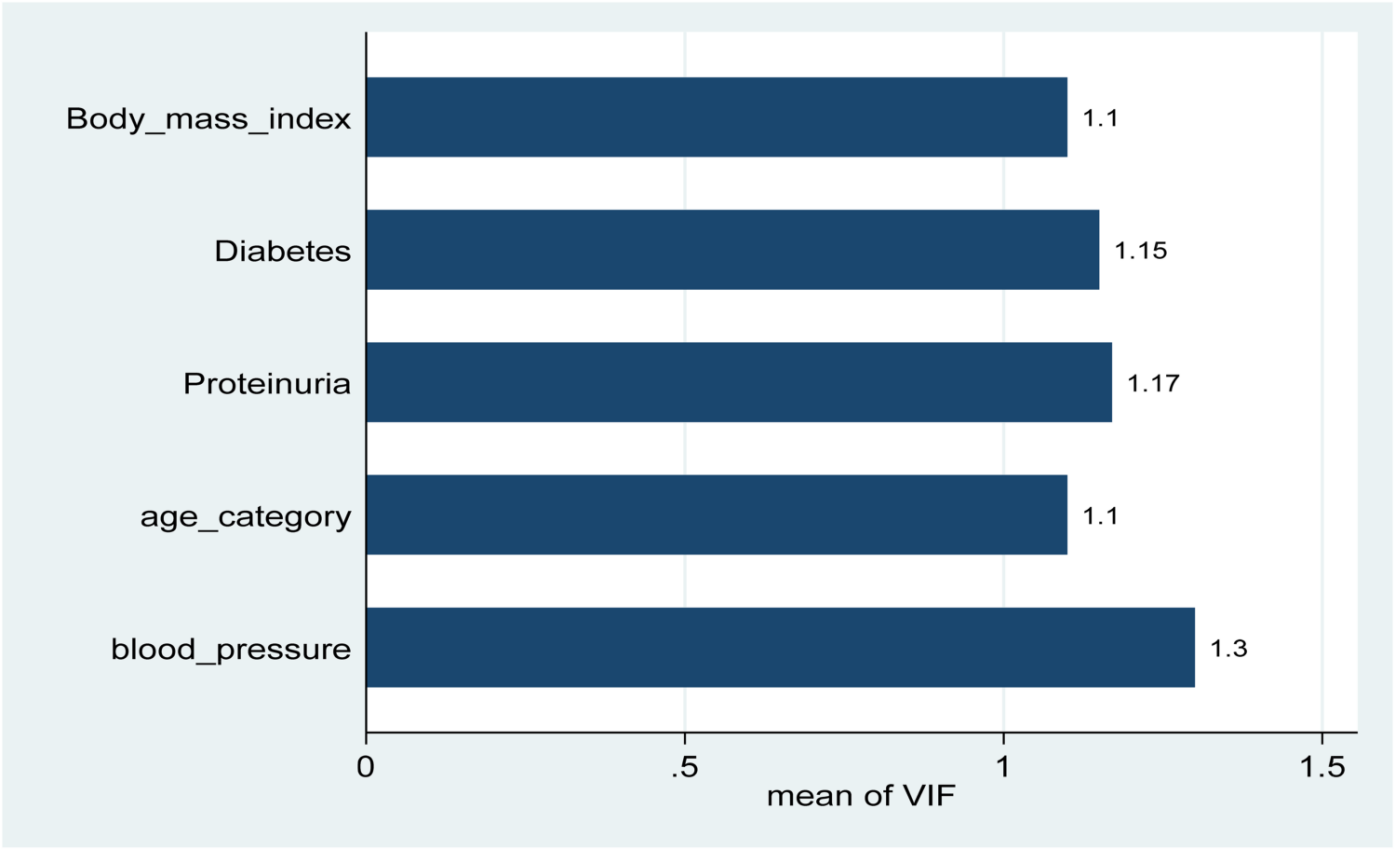
Variance inflation factors (VIF) for independent variables were included in the multivariable logistic regression model. All variables had VIF values ranged from 1.10 to 1.30 (mean VIF = 1.16), all well below the common threshold of 2.5, indicating no evidence of problematic multicollinearity.

Finally, overweight individuals were significantly more likely to have CKD, with an AOR of 3.37 (95% CI: 1.65–6.88; *P* = 0.00), indicating that maintaining a healthy weight is crucial in preventing CKD among hypertensive patients. These findings emphasize the prevalence and impact of hypertension on CKD, particularly the importance of age, proteinuria, diabetes, blood pressure control, and BMI as critical factors in the management and prevention of chronic kidney disease among adults in Burao (Table 3). Before conducting the multivariate analysis, we assessed the multicollinearity among the independent variables. All VIF values ranged from 1.10 to 1.30 (mean VIF = 1.16), indicating no evidence of problematic multicollinearity (Figure 1).

**Table 3:** Factors associated with chronic kidney diseases among adult hypertensive patient in Burao general hospital in Burao, Somaliland, 2024.

Variable name	Responses	Chronic kidney disease		COR (95% CI)	*P*-value	AOR (95% CI)	*P*-value
		Yes	No				
Age	20–26	13	6	Ref		Ref	
	27–33	19	19	2.16 (0.68, 6.89)	0.19	2.20 (0.66, 7.26)	0.19
	34–41	36	38	2.28 (0.78, 6.66)	0.12	2.20 (0.67, 7.22)	0.19
	42–48	19	33	3.76 (1.22, 11.53)	0.02*	3.39 (0.99, 11.54)	0.05*
	49–55	17	23	1.72 (0.92, 9.28)	0.06	2.50 (0.69, 9.06)	0.16
	>56	20	19	2.05 (0.64, 6.52)	0.22		
Sex	Female	61	77	0.76 (0.47, 1.24)	0.28		
	Male	63	61	Ref			
Marital statues	Single	13	10	Ref			
	Married	96	111	1.50 (0.63, 3.58)	0.35		
	Divorced	14	12	1.11 (0.36, 3.44)	0.85		
	Widow	1	5	6.49 (0.65, 64.82)	0.11		
Types of hypertensions	Systolic	92	97	Ref			
	Mixed	32	41	1.21 (0.70, 2.09)	0.48		
Classification of hypertension	Primary	86	84	Ref			
	Secondary	38	54	1.45 (0.87, 2.42)	0.15		
Number of drugs	One	76	85	Ref			
	Two	30	42	1.25 (0.71, 2.19)	0.43		
	Three and above	18	11	0.54 (0.24, 1.22)	0.14		
Proteinuria	Yes	86	26	Ref		Ref	
	No	38	112	9.74 (5.49, 17.28)	<0.001*	10.72 (5.74, 20.04)	<0.001*
Diabetes mellitus	Yes	56	46	Ref			
	No	67	92	9.74 (5.49, 17.29)	<0.001*		
Dyslipidemia	Yes	40	67	Ref			
	No	84	92	1.45 (0.84, 2.499)	0.17		
Blood pressure	Controlled	95	124	Ref		Ref	
	Uncontrolled	28	12	0.32 (0.15, 0.67)	<0.001*	0.22 (0.09, 0.53)	<0.001*
Body mass index	Normal	103	97	Ref		Ref	
	Overweight	21	41	2.07 (1.14, 3.75)	0.17	3.37 (1.65, 6.88)	<0.001*

NB: *indicates there is statistically significant association, *P* ≤ 0.05.

## 4 Discussion

This study examined the prevalence and impact of hypertension on chronic kidney disease (CKD) among adults in Burao General Hospital, Somaliland, revealing that 52.67% (95% CI: 46.6%–58.7%) of hypertensive patients also had CKD. Significant factors associated with CKD include age, proteinuria, diabetes, blood pressure, and body mass index (BMI). In patients aged 34–41 showing a higher likelihood of developing CKD. Notably, the observed association for the 34–41 age group, while statistically significant at *P* = 0.05, had a wide confidence interval crossing 1 (0.99–11.54), suggesting considerable uncertainty around this estimate. This finding emphasizes the need for cautious interpretation and further studies with larger sample sizes to confirm this finding.

Proteinuria emerged as a highly significant risk factor (AOR = 10.72; 95% CI: 5.74–20.04; *P* < 0.001), underscoring its central role as both a marker and contributor to renal damage. Diabetes mellitus was also strongly associated with CKD (AOR = 9.74), consistent with extensive literature linking chronic hyperglycemia to progressive renal injury (24–26). Overweight individuals had markedly higher odds of CKD (AOR = 3.37; 95% CI: 1.65–6.88; *P* < 0.001) (27, 28), reflecting the contribution of elevated BMI to kidney disease risk. These findings underscore the importance of identifying and managing these associated factors, which may help reduce the burden of CKD, although causality cannot be inferred owing to the cross-sectional nature of the study.

The prevalence of CKD observed in this study aligns with the findings from other regions, although the figures vary significantly. Studies in Ethiopia reported a prevalence of 39% among hypertensive patients, whereas research in Tanzania reported a lower prevalence of 12.4% (23, 29). Conversely, a higher prevalence of 57.1% has been reported in Nigeria among similar populations (30), indicating substantial regional differences that may reflect variations in healthcare access, screening practices, patient demographics, and genetic factors.

Blood pressure control was statistically significant in relation to CKD outcomes. Patients with uncontrolled hypertension were significantly less likely to be free from CKD, with an adjusted odds ratio of 0.22 (95% CI: 0.09–0.53; *P* < 0.001), indicating that uncontrolled blood pressure markedly increased the risk of developing CKD. This finding reinforces the critical importance of achieving and maintaining optimal blood pressure targets to mitigate kidney damage in hypertensive individuals, consistent with previous evidence that prolonged elevated blood pressure accelerates nephron loss and glomerular injury (1, 31, 32). Hypertension contributes to CKD progression by inducing increased intraglomerular pressure and promoting glomerulosclerosis, leading to long-term impairment of the filtration capacity. Diabetes mellitus accelerates kidney damage through chronic hyperglycemia, causing thickening of the glomerular basement membrane and mesangial expansion (33, 34). Obesity further exacerbates this risk by inducing hyperfiltration, activating pro-inflammatory pathways, and contributing to insulin resistance (35, 36).

Socioeconomic factors likely play a substantial role in shaping these outcomes. Many participants came from low-income settings with limited access to routine healthcare services, potentially delaying the diagnosis and management of hypertension and diabetes. Lower health literacy, economic constraints affecting diet and medication adherence, and lifestyle limitations may have compounded these risks.

These findings reinforce the existing evidence that emphasizes the critical importance of integrating chronic disease management, routine screening for kidney dysfunction, and broader public health interventions. Targeted strategies addressing both medical and socioeconomic factors are essential to effectively mitigate the burden of CKD in low-resource settings.

## 5 Conclusion

This study investigated the prevalence and impact of hypertension on chronic kidney disease (CKD) among adults attending Burao General Hospital in Somaliland. The findings revealed a significant prevalence of CKD (52.67%) among patients with hypertension, underscoring the critical relationship between uncontrolled hypertension and CKD development. The key risk factors identified included age, diabetes, proteinuria, and body mass index. These results highlight the urgent need for strengthened hypertension management and monitoring of associated factors to potentially reduce the burden of CKD in this population; however, causality cannot be established owing to the study design.

## 6 Recommendations

Several recommendations should be implemented to effectively address the prevalence of hypertension and its impact on chronic kidney disease (CKD) in Burao City. First, the Burao General Hospital should develop and implement comprehensive hypertension management programs aimed at the early detection and effective management of hypertension. These programs will help prevent CKD progression in at-risk populations. In addition, increasing public awareness through community health education campaigns is essential. Such initiatives can inform the local community about the risks associated with hypertension and CKD, highlighting the importance of regular checkups and lifestyle modifications.

Establishing routine screening protocols for CKD in hypertensive patients is another critical step. This would include regular urine tests for proteinuria and the assessment of kidney function, which can facilitate early intervention and management. Integrating lifestyle modification strategies into patient management plans is vital. Encouraging healthy eating, regular physical activity, and smoking cessation can significantly improve the health outcomes of individuals with hypertension.

Finally, collaborating with local health authorities will be crucial in improving access to healthcare services and resources for managing hypertension and CKD. By working together, healthcare providers can enhance service delivery, ensuring that patients receive the necessary support and resources to effectively manage their conditions. These recommendations, supported by existing literature, aim to mitigate the impact of hypertension on kidney health in the community.

## 7 Strengths and limitations

### 7.1 Strengths

This study had several notable strengths. It employs structured questionnaires and clinical data, which provide a robust dataset for analyzing factors associated with chronic kidney disease (CKD). Focusing specifically on adults in Burao City addresses a critical research gap, offering tailored insights into the local context. Additionally, the use of multivariate logistic regression allows for the identification of significant predictors of CKD, enhancing our understanding of the relationship between hypertension and kidney health.

### 7.2 Limitations

However, this study had several limitations. Its cross-sectional design restricts its ability to establish temporal or causal relationships between hypertension, associated factors, and CKD. Therefore, all observed relationships should be interpreted strictly as an association. Additionally, BMI and blood pressure data were only available in categorical form, which limited our ability to report the mean values or analyze these variables as continuous outcomes. Reliance on self-reported data may introduce a recall bias, potentially affecting data accuracy. The findings also have limited generalizability because of the specific population studied in Burao City. Finally, the exclusion of non-respondents could result in selection bias, further impacting the representativeness of the sample.

## Data Availability

The raw data supporting the conclusions of this article will be made available by the authors, without undue reservation.
